# Molecular remodeling in comorbidities associated with heart failure: a current update

**DOI:** 10.1007/s11033-024-10024-7

**Published:** 2024-10-26

**Authors:** Sandeep Appunni, Muni Rubens, Venkataraghavan Ramamoorthy, Anshul Saxena, Peter McGranaghan, Atulya Khosla, Mayur Doke, Sandra Chaparro, Javier Jimenez

**Affiliations:** 1 Government Medical College, Kozhikode, Kerala India; 2 Miami Cancer Institute, Baptist Health South Florida, Miami, FL USA; 3 Herbert Wertheim College of Medicine, Florida International University, Miami, FL USA; 4 Universidad Espíritu Santo, Samborondón, Ecuador; 5 Center for Advanced Analytics, Baptist Health South Florida, Miami, FL USA; 6 Semmelweis University, Budapest, Hungary; 7 William Beaumont University Hospital, Royal Oak, MI USA; 8 University of Miami, Miami, FL USA; 9 Miami Cardiac & Vascular Institute, Baptist Health South Florida, Miami, FL USA; 10 Advance Heart Failure and Pulmonary Hypertension, South Miami Hospital, Baptist Health South, Miami, FL USA; 11 Department of Internal Medicine and Cardiology, Charité-Universitätsmedizin Berlin Corporate Member of Freie Universität Berlin and Humboldt Universität zu Berlin, Augustenburger Platz 1, 10117 Berlin, Germany

**Keywords:** Heart failure, Obesity, Diabetes mellitus, Systemic hypertension, Pulmonary hypertension, Coronary artery disease, Hypercholesteremia, Chronic kidney disease, Atrial fibrillation

## Abstract

Recent advances in genomics and proteomics have helped in understanding the molecular mechanisms and pathways of comorbidities and heart failure. In this narrative review, we reviewed molecular alterations in common comorbidities associated with heart failure such as obesity, diabetes mellitus, systemic hypertension, pulmonary hypertension, coronary artery disease, hypercholesteremia and lipoprotein abnormalities, chronic kidney disease, and atrial fibrillation. We searched the electronic databases, PubMed, Ovid, EMBASE, Google Scholar, CINAHL, and PhysioNet for articles without time restriction. Although the association between comorbidities and heart failure is already well established, recent studies have explored the molecular pathways in much detail. These molecular pathways demonstrate how novels drugs for heart failure works with respect to the pathways associated with comorbidities. Understanding the altered molecular milieu in heart failure and associated comorbidities could help to develop newer medications and targeted therapies that incorporate these molecular alterations as well as key molecular variations across individuals to improve therapeutic outcomes. The molecular alterations described in this study could be targeted for novel and personalized therapeutic approaches in the future. This knowledge is also critical for developing precision medicine strategies to improve the outcomes for patients living with these conditions.

## Introduction

Heart failure is a terminal cardiac event in which the structural and functional capacity of the myocardium is compromised resulting in inadequate systemic perfusion [1]. Heart failure involves substantial healthcare burden in terms of decreased life expectancy, poor quality of life, and increased medical expenditures. Due to advanced treatment strategies, the outcomes of heart failure are steadily improving. However, the prevalence of heart failure is on the rise due to the aging population and better survival rates.

Heart failure is a complex and heterogeneous disease that is associated with several comorbidities. These comorbidities can significantly impact the course of heart failure and contribute to poor outcomes. In the recent years, advances in genomics and proteomics have unlocked newer opportunities for understanding the underlying molecular mechanisms of comorbidities in heart failure, thus paving way for more personalized approaches to prevention and treatment [2]. Studies have identified genetic variants that are associated with both heart failure and related comorbidities, suggesting that there may be shared molecular pathways underlying these associations [3]. By understanding these pathways, researchers could develop more targeted and personalized approaches for prevention and treatment that consider the individual’s unique genetic and biological profile. In addition to identifying shared genetic variants and pathways, genomic research also highlights the importance of epigenetic and non-coding genetic variants in the development and progression of comorbidities in heart failure [4]. Genomic research could also contribute to the development of newer diagnostic and prognostic tools for identifying patients at increased risk of developing comorbidities or experiencing poor outcomes in heart failure.

Heart failure is caused by several pathophysiological processes, including the activation of the neurohormonal systems, oxidative stress, dysfunctional calcium handling, poor energy utilization, mitochondrial dysfunction, and inflammation [5]. Typically, myocardial loss leads to increased myocardial remodeling and is associated with heart failure with reduced ejection fraction (HFrEF) [5]. Contrarily, patients with comorbidities such as diabetes mellitus, obesity, and hypertension are more likely to get heart failure with preserved ejection fraction (HFpEF), resulting from the development of a microenvironment characterized by persistent chronic inflammation [5]. Since comorbidities associated with heart failure have an important role in worsening cardiac pathophysiology, in this narrative review, we aim to update the recent advances in molecular changes associated with heart failure and comorbidities. We included obesity, diabetes mellitus, systemic hypertension, pulmonary hypertension, coronary artery disease, hypercholesteremia and lipoprotein abnormalities, chronic kidney disease, and atrial fibrillation because these are the most prevalent comorbidities in heart failure and molecular alterations associated with these conditions could be useful targets for future therapeutic approaches.

## Methods

This study is a narrative review of the association between comorbidities and heart failure. We searched the electronic databases, PubMed, Ovid, EMBASE, Google Scholar, CINAHL, and PhysioNet for articles without time restriction.

### Common comorbidities associated with heart failure

Even though heart failure treatment has advanced over the years, the burden of associated comorbidities remains high and greatly influences therapeutic outcomes [6]. Comorbidities are identified in more than 70% of heart failure patients and have an independent effect on morbidity and mortality [6].

Obesity is a major comorbidity associated with heart failure and requires early prevention measures [6]. The relationship between heart failure and diabetes is bidirectional. The pathogenesis of diabetes in heart failure has been attributed to increased adrenergic tone, decreased insulin sensitivity, micro and macrovascular changes, congestion in the pancreas, and the effects of medications [6]. On the other hand, type 2 diabetes mellitus, which is an important comorbidity among heart failure patients, increases the risk of new-onset heart failure, particularly when patients require insulin therapy or have other concomitant comorbidities [7]. Systemic hypertension is another comorbidity, which is often associated with left ventricular hypertrophy (LVH) and remodeling [7]. Both hypertension and heart failure are associated with higher body mass index (BMI) [7], sleep disorders, depression, and iron deficiency anemia [7]. Other common comorbidities associated with heart failure include systemic hypertension, pulmonary hypertension, coronary artery disease, hypercholesteremia and lipoprotein abnormalities, chronic kidney disease, and atrial fibrillation [7]. Therapeutic management of heart failure should also ensure adequate control over these comorbidities for better clinical outcomes. These comorbidities are also associated with molecular alterations in gene expressions which could occur due to genetic and environmental factors.

### Obesity

Obesity is an important risk factor for heart disease. Higher BMI is associated with greater risk of developing heart failure and associated morbidity and mortality [8]. High output heart failure is common among obese individuals and is characterized by increased cardiac output, decreased vascular resistance, and enhanced oxygen consumption [9]. Obesity is associated with increased risk for developing HFpEF [9]. Genes upregulated in HFpEF enrich pathways related to mitochondrial adenosine triphosphate synthesis/electron transport, while genes downregulated are related to endoplasmic reticulum stress, autophagy, and angiogenesis [9]. Energy metabolism is variably impaired in heart failure depending upon the associated comorbidities. For example, obesity and diabetes mellitus increase mitochondrial oxidation, resulting in increased ATP synthesis, while mitochondrial oxidation is decreased in heart failure due to ischemia or hypertension [10]. This occurs due to transcriptional dysregulation in the expression of the genes coding for key enzymes involved in the metabolic processes such as glycolysis and mitochondrial oxidation in heart failure. In addition, alterations in the redox states of nicotinamide adenine dinucleotide (NAD) and downstream signaling contribute to post-translational epigenetic changes in the regulation of genes encoding for the enzymes involved in energy metabolism [10]. Obese individuals with metabolic syndrome are at a greater risk of developing HFpEF and exercise induced pulmonary hypertension. A study on obese ZSF-1 leptin-receptor knockout HFpEF rat models showed increased right ventricular systolic pressure at rest on treatment with SU5416, which increases pulmonary arterial pressure. Among these rat models, introduction of exercise exacerbated pulmonary arterial hypertension. These features were associated with increased reactive oxygen species (ROS) and decreased soluble guanyl cyclase beta 1 (sGCβ1) in the smooth muscle cells of the pulmonary arteries [11]. ROS decreased sGCβ1 by upregulating miR-193b, which degraded nuclear factor Y α subunit (NFYA). However, upregulation of NFYA expression through adeno-associated virus 6 delivered to the pulmonary arteries decreased the effects of pulmonary hypertension among obese ZSF-1 rats [11]. Likewise, pharmacological inhibition of cyclic GMP sensitive phosphodiesterase 9 has also shown to decrease fat deposition, while improving left ventricular ejection fraction in association with upregulation of peroxisome proliferator-activated receptor alpha (PPAR-α) [11]. Another genetic alteration characteristic of HFpEF is upregulation of Forkhead box protein O1 (FoxO1), which is associated with downregulation of spliced form of the X-box-binding protein 1 (Xbp1s). Increased activity of FoxO1 is associated with cardiomyocyte steatosis, which decreases cardiac efficiency in HFpEF. In experimental mice, downregulation of FoxO1 or overexpression of Xbp1s decreased lipid accumulation in cardiomyocytes by modulating the unfolded protein response in HFpEF [12]. Xbp1s triggers ubiquitination and proteasomal degradation of FoxO1, which is mediated through the activation of E3 ubiquitin ligase, STIP1 homology and U-box-containing protein 1 (STUB1) in the cardiac myocytes [12]. Wang et al. showed that high fructose diet in vivo aggravated inflammation and cardiac dysfunction [13]. After 12 weeks of feeding high fructose diet, the levels of monocyte chemoattractant protein-1 (MCP-1) increased among mouse models in vivo, and triggered the expression of pro-fibrotic genes, inflammatory mediators, and cardiac infiltration of macrophages.

Although there are many molecular targets that could be focused on managing obesity and heart failure, as described above, there are very few that have been used therapeutically. For example, a novel drug, sodium-glucose transport protein-2 (SGLT-2) inhibitor, dapagliflozin, aims at a similar target. Using transcriptional and proteomic analyses, Almengló et al. reported increased levels of adhesion molecules in neutrophils among obese patients [14]. This study showed that the levels of adhesion molecule CD11b were higher among patients who had both obesity and HFpEF. The mediators secreted by adipose tissue upregulated the adhesion protein, while SGLT-2 inhibitor, dapagliflozin, downregulated it. Thus, SGLT-2 inhibitors are used in HFpEF due to their effects on reducing visceral adiposity and decreasing cardiac inflammation through lowering the levels of inflammasomes. Lin et al. selectively studied the benefits of dapagliflozin treatment on the effects of inflammation induced by palmitic acid on cardiomyocytes in vitro and in vivo [15]. Palmitic acid induced inflammation and had direct toxic effects on the cardiomyocytes resulting in cardiomyopathy. Cultured cardiomyocyte pretreated with dapagliflozin were subsequently exposed to palmitic acid. Dapagliflozin pretreatment decreased hypertrophy, fibrosis, and apoptosis that was induced by palmitic acid treatment. In H9C2 line of embryonic rat cardiomyocytes, dapagliflozin showed a protective role through inhibition of palmitic acid-activated MAPK/AP-1 pathway as investigated through transcriptome analysis. Dapagliflozin showed direct binding to NHE1 as determined by surface plasmon resonance analysis. This shows that NHE/MAPK/AP-1 was significantly influenced by dapagliflozin and decreased the cardiotoxic effects of palmitic acid. In the same study, in vivo experiments on mice fed with high fat diet showed that dapagliflozin treatment reduced cardiac inflammation and high fat diet induced cardiac dysfunction by suppressing the MAPK/AP-1 pathway and cardiac remodeling. This suggests that SGLT-2 inhibitors have additional direct cardioprotective action in addition to the canonical effects resulting from glycemic control. Conjunction of SGLT-2 inhibitors with glucagon-like peptide-1 receptor agonists (GLP-1RA) might have promising benefits in reducing cardiometabolic toxicity. Based on a large clinical trial finding, GLP-1 RA had the greatest beneficial effect on HbA1c reduction, weight loss, and prevention of atherosclerotic cardiovascular disease outcomes, including stroke, while SGLT-2 inhibitors had significant effects on decreasing heart failure hospitalizations and progression of chronic kidney disease (CKD) [16]. Adamson et al. reported that obese patients on dapagliflozin experienced greater symptom relief and better cardiovascular outcomes throughout the BMI spectrum, compared to placebo [17]. Another study by Oyama et al. showed that though relative risk reduction in cardiovascular and renal outcomes due to dapagliflozin were similar across the BMI spectrum, absolute risk reduction in obesity-related outcomes such as heart failure hospitalizations and atrial fibrillation/flutter were significantly higher among obese patients with concomitant type 2 diabetes [18].

Empagliflozin, another SGLT2 inhibitor, showed significant improvement in insulin signaling in cardiac tissue and decreased cardiac hypertrophy and ischemic events among obese insulin resistant mice. Empagliflozin treatment demonstrated cardioprotective effects by improving myocardial metabolic flexibility and enhanced cardiac specific substrate utilization demonstrated through administration of [U^13^C]-glucose among fasting mice [19]. SGLT-2 inhibitors have protective effects on cardiomyocyte by other non-glycemic control measures as well. For example, ipragliflozin treatment among male DahlS.Z-Lepr^fa^/Lepr^fa^ non-diabetic obese rats reduced systolic blood pressure, interventricular septal thickness and left ventricular mass [20]. Ipragliflozin altered the microRNA profile related to left ventricular hypertrophy and heart failure while also decreasing the circulatory cytokine levels in vivo. Understanding the molecular alterations associated with obesity and heart failure could help in developing effective therapeutic interventions.

### Diabetes mellitus

Diabetes is an independent risk factor for heart failure. Both diabetes and heart failure adversely influence one another. For example, a Mendelian randomization study by Mordi et al. showed that type 2 diabetes and heart failure had bidirectional effects and directional pleiotropy [21]. The enhanced oxidative stress and metabolic alterations in diabetes mellitus resulted in DNA damage leading to diabetic cardiomyopathy. Ataxia-telangiectasia mutated kinase (ATM) protein is produced in response to this DNA damage, and impedes cell cycle, senses redox changes, and promotes DNA repair [21]. ATM production is dysregulated in insulin resistance and diabetes mellitus and increases the risk of cardiomyopathy induced heart failure [21]. Another modifiable group of targetable proteins includes the PI3K family of kinases which are involved in diabetes mellitus and heart failure. PI3K isoforms such as PI3K-α has a protective role against diabetes mellitus induced heart failure, while the inhibition of oxidative stress generating PI3K-γ has protective effects against heart failure [22]. Activation of the PI3K/AKT/mTOR/HIF-1α signaling pathway following miR-372-3p knock down impedes the development of diabetic cardiomyopathy in vivo, which in turn reduces the risk for heart failure [23]. MiR-372-3p knock down also suppresses oxidative stress and enhances the levels of NOX2 and NOX4, which in conjunction with activation of PI3K signaling cascade initiates angiogenesis in vivo and impedes the progression of diabetic cardiomyopathy [23]. In another study that relates diabetes and heart failure, Wu et al. showed that dysfunction of endothelial SIRT6 increased fatty acid uptake across endothelium among an experimental model of diabetic mice with HFpEF [24]. Deficiency of SIRT6 upregulates the expression of fatty acid transporters, thus increasing fatty acid uptake and lipid accumulation [25]. SIRT6 represses endothelial PPAR-γ expression by inducing histone deacetylation of H3 lysine 9 residues around the promoter region of PPAR-γ [24]. This SIRT6 mediated PPAR-γ reduction results in the inhibition of endothelial fatty acid uptake. Genetic and pharmacological restoration of SIRT6 reverses the effect of fatty acid transport. This suggests that modulating SIRT6 expression in heart failure that is associated with diabetes could be a potential target for decreasing cardiac lipid accumulation. Likewise, in another study, Li et al. showed that microRNA miR-320 was upregulated in patients with diabetic cardiomyopathy [26]. miR-320 targets and transcriptionally upregulates fatty acid translocase expression. This is associated with alteration of blood lipid homeostasis and causes enhanced cardiac lipid uptake and cardiomyocyte lipotoxicity.

Among experimental type 2 diabetes mouse models, Veitch et al. showed that the expression of miR-30d-5p and miR-30e-5p increased in circulating extracellular vesicles [27]. These are markers of coronary microvascular endothelial cell dysfunction and correlate with echocardiographic features of diastolic dysfunction [27]. Endothelial dysfunction was associated with increased DNA damage and senescence as well as increased levels of ROS and lipid peroxidation due to increased levels of fatty acid beta-oxidation in the endothelial cells. Among type 2 diabetes experimental mouse models, decreased endothelial nitric oxide synthase (eNOS) activity in the endothelial cells increases the risk for coronary microvascular complications, thus potentially increasing the risk for developing HFpEF. In another study among experimental mice models with high fat diet induced diabetes mellitus, Mu et al. showed that activation of mitophagy and clearance of defective mitochondria was associated with improved cardiac functions [28]. Inhibition of PTEN-induced kinase 1 (PINK1)/Parkin-mediated mitophagy due to BRD4 upregulation causes damaged mitochondria to accumulate in cardiomyocytes, thus impairing cardiac shape and function among diabetic mouse hearts. BRD4 suppression by JQ1 enhanced mitochondrial activity restored the cardiac structure and function among diabetic mice. These findings show that genetic alterations in hyperglycemic state potentiate pathological responses and impede the function of cardiomyocytes.

Wang et al., in a bioinformatics study reported 233 upregulated and 3 downregulated genes that were commonly involved in heart failure and diabetes mellitus [29]. In this study, functional enrichment analysis of the differentially expressed genes showed that the key modules were primarily involved in immunity. Similarly, protein-protein interactions identified a set of five hub genes which include *SYK*, *SELL*, *RAC2*, *TLR8* and *ITGAX* which are involved in heart failure and diabetes. Likewise, using bioinformatics platform, Guo et al. identified five potential immune biomarkers in microarray datasets from diabetic cardiomyopathy, which include proteosome subunit beta type-8 (*PSMB8*), nuclear factor kappa B1 (*NFKB1*), albumin (*ALB*), endothelin 1 (*EDN1*), and estrogen receptor 1 (*ESR1*) [30]. The altered expression of these genes was validated on experimental models and *END1* showed significantly higher gene expression among patients with diabetic cardiomyopathy, compared to healthy controls. Among patients with diabetes mellitus associated heart failure, Xu et al. also identified key network targets, biological pathways, and processes that are related to immunological functions and targeted by the phytoestrogen, calycosin [31]. Using molecular docking methodology, Xu et al. identified mitogen-activated protein kinase-1 (MAPK1), Arrestin Beta 1 (ARRB1), and Abelson Murine Leukemia Viral Oncogene Homolog 1 (ABL1) as potential pharmacological targets of calycosin among these patients These findings show that alterations occurring in the expression of immune markers, related networks, and functional processes are impaired in diabetes mellitus and heart failure and needs further exploration from a therapeutic perspective.

Therapeutic interventions targeting specific receptors that are dysregulated in type 2 diabetes could alter the outcomes of heart failure in diabetes. For example, in a randomized controlled trial, Petrie et al. showed that dapagliflozin decreased cardiovascular mortality and morbidity among heart failure patients with type 2 diabetes [32]. Dapagliflozin is used for the treatment of type 2 diabetes mellitus and decreases renal glucose reabsorption, thus inducing glucosuria [32]. However, SGLT-2 inhibitors are also important in comprehensive heart failure management due their cardioprotective effects such as diuresis, natriuresis, optimization of blood pressure levels, and improved cardiac metabolism [32]. SGLT-2 inhibitors also prevent cardiac remodeling, ischemia-reperfusion injury, hyperuricemia, oxidative stress, inflammation, and sympathetic nervous system activation, and increase vascular progenitor cells, vascular functionality, autophagy, and erythropoietin levels. These effects are achieved through modulation of molecular targets such as sodium/hydrogen exchanger, calmodulin-dependent protein kinase II, nucleotide-binding oligomerization domain leucine-rich repeat, tumor necrosis factor-alpha, and plasminogen activator inhibitor-1 [32].

Another class of drugs which could alter the outcomes of heart failure in diabetes is angiotensin receptor-neprilysin inhibitor combination. In heart failure, this drug combination acts through blocking the RAAS system. Khan et al. showed that administration of sacubitril/valsartan therapy was associated with significant improvements in cardiac remodeling and N-terminal pro-b-type natriuretic peptide (NT-proBNP) levels [33]. Wijkman et al. also reported that sacubitril/valsartan treatment lowered HbA1c and decreased the requirement for insulin therapy among patients with concomitant heart failure and diabetes [34]. However, the mechanism of the effects of this drug combination among heart failure patients with diabetes remains unclear. In a study by Ge et al. among streptozotocin-induced diabetic mice and H9C2 cardiomyoblast cell line exposed to high glucose, sacubitril/valsartan inhibited the nuclear transfer of NF-κB and JNK/p38MAPK phosphorylation. Through these mechanisms, sacubitril/valsartan decreased cardiac inflammation, oxidative stress, and apoptosis and improved diabetic cardiomyopathy. These findings suggest that heart failure and diabetes have common molecular pathways that could be targeted for therapeutic interventions.

### Systemic hypertension

Systemic hypertension is an important comorbidity associated with heart failure due to its high prevalence in the general population. Chronic pressure overload leads to left ventricular hypertrophy, ventricular fibrosis, diastolic dysfunction, decreased left ventricular filling pressure, and eventually diastolic heart failure. Several changes occurring at the molecular and cellular levels contribute to the pathophysiological changes observed in hypertension associated heart failure. For example, Shou et al. created HFpEF model rats by inducing a hypertensive state and observed that defective mitochondrial fission process led to mitochondrial dysfunction [35]. HFpEF rats had significant alteration in genes involved in tricarboxylic acid (TCA) cycle, ATP production, and mitochondrial fission. This study showed that, in HFpEF, there was downregulation of PTEN-induced kinase 1 (PINK1) which diminished Drp1^S616^ phosphorylation and led to its mitochondrial localization, thereby resulting in impaired mitochondrial fission [35]. Enhancing PINK1 mediated Drp1^S616^ phosphorylation to increase mitochondrial fission and improving mitochondrial functions could have potential therapeutic implications in hypertension induced HFpEF. Among spontaneous hypertensive rats, which are models for primary hypertension and left ventricular hypertrophy (LVH), Bednarski et al. showed that there were alterations in lipid metabolites which accumulated in the left ventricle [36]. This study showed that triacylglycerol levels were higher while phosphorylated adenosine monophosphate-activated protein kinase (AMPK) and PPAR-α protein contents were lower in cardiomyocytes of the left ventricle. These changes suggest that β-oxidation and lipolysis were diminished in cardiomyocytes, and energy utilization processes were inefficient in hypertension induced LVH, resulting in cardiac dysfunction. Cardiosphere-derived cells (CDC) are stem cells that show beneficial effects on tissue regeneration when introduced by intramyocardial injection or intracoronary infusion [37]. They achieve this process by inducing proliferation of cardiomyocytes and activation of native stem cells. In hypertensive HFpEF rats, CDCs decreased oxidative stress and showed anti-inflammatory effects following intra-coronary injections [38]. CDCs induced vasodilation by upregulating eNOS activity. In addition, exosome vesicular products of CDCs decreased monocyte activity by suppressing the functions of vascular cell adhesion molecule 1 (VCAM-1) such as monocyte adhesion and transmigration [38].

In a genomic study using microarray data of rat model with hypertensive cardiac hypertrophy, Zhang et al. showed that 348 genes were differentially expressed [39]. These genes were involved in biological processes such as organization of collagen fibrils, effects of transforming growth factor-beta (TGF-β) on cellular function, extracellular matrix (ECM)-receptor interactions, and oxytocin signaling pathway [39]. This study also showed that two upregulated genes, *Eln* and *Tgfb3* were positively correlated with disease severity in hypertension-induced cardiac hypertrophy [39]. *Eln* expresses elastin, which is an important extracellular matrix protein that provides elasticity and resilience to the tissues, while *Tgfb3* expresses one of the three types of TGF-β isoforms which regulates the pathogenesis of fibrosis [40]. Recent evidences suggest that polymorphism in *WNT3A* rs752107(C > T) which affects the Wnt/β-catenin signaling pathway increases the risk of essential hypertension and associated cardiovascular events such as heart failure and ischemic stroke [41]. Compared to *WNT3A* rs752107 CC genotype, TT variant has higher risk (139% TT vs. 48% CC) for essential hypertension, and the risk of heart failure increases by 58% among individuals carrying T allele. Future studies should focus on regulating Wnt/β-catenin signaling pathways at various levels such as regulating the expression of *CTNNB1* which encodes for β-catenin for controlling hypertension related heart failure. Hypertension related LVH and heart failure involve many underlying molecular processes that are associated with cardiomyocyte contractility and functions. For example, a study that included tissue culture and in vivo procedures showed that activin A levels were upregulated in age-related conditions such as hypertension, atherosclerosis, and heart failure [42]. This study also showed that overexpression of activin A induced by gene transfer in vivo impaired cardiac contractility and induced myocardial atrophy. Activin A mediated activation of cardiomyocyte resulted in impaired diastolic calcium flux and was associated with downregulation of calcium homeostasis regulatory genes such as *SERCA2*, *RYR2*, and *CACNB2* [42]. Antihypertensive medications such as hydrochlorothiazide, fasudil, and spironolactone profoundly remodel the altered molecular processes in hypertension induced heart failure. For example, in a study done among deoxycorticosterone acetate salt induced hypertensive rats, hydrochlorothiazide significantly decreased the activation of rho-associated protein kinase (ROCK), decreased the levels of profibrotic proteins (Col-I, Col-III and TGF-β1), decreased pro-remodeling molecules (TGF-β1, CTGF, MCP-1 and PAI-1), and decreased the expression of oxidative stress related genes (9gp91phox and p22phox) [43]. These effects were associated with decreased myocardial hypertrophy and fibrosis and delayed pathological remodeling. These altered molecular processes associated with systemic hypertension and heart failure could be targeted for future interventions.

### Pulmonary hypertension

Pulmonary hypertension (PH) is defined as mean pulmonary artery pressure > 20 mm Hg and is categorized into clinical subtypes based on etiology, pathophysiology, and therapeutic approaches [44]. Pulmonary arterial hypertension (PAH) is one of the five subtypes of PH and is characterized by remodeling of pulmonary arteries leading to mean pulmonary artery pressure of > 20 mm Hg, pulmonary artery wedge pressure of ≤ 15 mm Hg, and pulmonary vascular resistance of 3 Wood units [44]. PAH that is detected early in life is associated with genetic factors and has complex and multifactorial etiologies [44]. Current research shows that approximately 25–30% percent of individuals with idiopathic PAH have a hereditary cause [45]. For patients with early onset idiopathic PAH, genetic testing for associated genes such as *ACVRL1*, *BMPR2*, *CAV1*, *ENG*, and *KCNK3* could assist in early diagnosis and prompt treatment [46]. *BMPR2* is the most commonly involved gene associated with familial and idiopathic PAH and has more than 300 associated mutations [46]. Recently, Du et al. showed that patients having congenital heart disease (CHD) associated PH (CHD-PH) also have *BMPR2* mutations [47]. Defective BMPR2 signaling was associated with compromised cardiac function and impaired calcium transients that were detected using confocal microscopy. The downstream effector protein of *BMPR2* functions as inhibitors of DNA binding proteins (ID), ID1 and ID3. Cardiomyocytes differentiated from induced pluripotent stem cells (iPSC) of CHD-PH patients showed decreased smad1/5 phosphorylation and ID1/ID3 expression [47]. Cardiomyocyte specific ID1/ID3 knockout mice developed features of minimal PH and remodeling of the pulmonary vasculature. In the same study, *ID1* and *ID3* double knockout mesodermal cells derived from CHD-PH patients showed reduced BMPR2/ID signaling [47]. These findings show that altered expression of cardiomyocyte specific genes play an important role in the pathogenesis of PH.

PAH progressively deteriorates right sided cardiac function and if not treated could progress to right heart failure, and eventually left heart failure. Right ventricle in comparison to left ventricle expresses higher levels of GATA Binding Protein 4 (GATA4), which is a transcription factor that modulates cardiac hypertrophy [48]. Right ventricular stress stimulated by hypoxia induced PH upregulates GATA4 expression through the binding of CCAAT-binding factor/nuclear factor-Y (CBF/NF-Y) to CCAAT box in the right ventricular myocytes, thereby triggering hypertrophy [48]. Mammalian target of rapamycin (mTOR) is another important pathway in the pathogenesis of PH. A study by Houssaini et al. showed that activation of mTOR complex 1 (mTORC1) and mTORC2 in the pulmonary artery smooth muscle cells induced pulmonary vasculature remodeling in PH, and was effectively inhibited by rapamycin [49]. However, in a recent study among old mice, McNair et al. showed that rapamycin mediated inhibition of mTOR was unable to decrease right ventricular remodeling triggered by hypobaric hypoxia induced PH, and eventually led to right ventricular failure [50]. Further studies should focus on the interaction between mTOR, hypoxia, and remodeling to better understand the underlying molecular mechanisms.

Recent advancements have shown a substantial genomic basis for both PH and right ventricular failure. A study by Cao et al. found that gene expressions were altered for 169 long non-coding RNA (lncRNA) and 898 mRNA among Sprague-Dawley rats that were injected with monocrotaline and lipopolysaccharide to precipitate combined PH and right ventricular failure [51]. A study by Legchenko et al. among U5416/hypoxia (SuHx) rat models showed that PH and right ventricular failure were associated with defects in intracellular metabolisms related to mitochondrial function and fatty acid oxidation. In the cardiomyocytes of these rat models, PPAR-γ agonist, pioglitazone, improved mitochondrial function and decreased intra-cardiomyocyte lipid accumulation (lipotoxicity) by modifying the epigenetic and transcriptional milieu [52]. In addition, the expression of pre-miR-197 and pre-miR-146b were upregulated in right ventricular failure, leading to downregulation of the genes associated with fatty acid oxidation such as carnitine palmitoyltransferase 1B (*Cpt1b*) and fatty acid binding protein 4 (*Fabp4*). These findings show that altered patterns of lncRNA, miRNA, and mRNA profiles and related molecular pathways are crucial in the pathogenesis of PH induced right ventricular failure and could be potential therapeutic targets.

PAH associated right ventricular dysfunction, morbidity, and mortality are substantially lower among women. This sexual dimorphism in the clinical outcomes could be attributed to the differences in the molecular mechanisms associated with hormones and other factors. For example, a study by Frump et al. showed that higher levels of 17β-estradiol (E2) among females inhibited PAH-induced RV injury by upregulating the pro-contractile and pro-survival peptide, apelin, through a BMPR2-dependent mechanism acting via the estrogen receptor-α (ER-α) [53]. Right ventricular homogenates from patients with right ventricular failure and experimental rats with right ventricular remodeling showed decreased expression of ER-α and apelin. E2 and ER-α upregulate BMPR2 expression in PAH induced right ventricular failure which potentially increased the production of apelin. These molecular mechanisms could be targeted for gender specific therapeutic approaches for PAH.

### Coronary artery disease

Coronary artery disease (CAD) is a major risk factor for heart failure. Even though atherosclerosis is the most common etiology, non-atherosclerotic causes are also associated with CAD. Common genetic variations such as single nucleotide polymorphisms (SNPs) are increasingly being linked to CAD and heart failure. Polymorphisms of genes associated with receptor for advanced glycation end-products (RAGE), IL-6, and SGLT-2 are related to CAD and heart failure [54–56]. For example, Falcone et al. found that AA phenotype of the *RAGE* gene was associated with decreased risk of ischemic heart failure, compared to non-ischemic related heart failure [54]. The presence of -374 T/A polymorphism in the *RAGE* gene was associated with the development of atherosclerotic coronary artery disease. In this study, patients with coronary artery involvement had significantly lower A allele and homozygous AA genotype, compared to those without coronary artery lesions. These findings suggested that the inheritance of AA genotype is possibly protective among patients with heart failure with atherosclerotic etiology. Kravchun et al. reported that polymorphism in the *IL6* gene with GG genotype had higher risks for heart failure among obese subjects with CAD. This finding demonstrated an association between heart failure and the presence of -174G allele and GG genotype in individuals with obesity and CAD [55]. Katzmann et al. using data from LUdwigshafen RIsk and Cardiovascular Health study found two SNPs in *SLC5A2* gene (which encodes for SGLT-2), rs11646054 and rs9934336, that were significantly associated with lower NT-proBNP among non-CAD, compared to CAD patients [56]. In the same study, using the UK Biobank data, Katzmann et al. four SNPs with minor allele frequency which were associated with CAD and heart failure. Besides SNPs, epigenetic alteration in gene expression was also found to be associated with CAD and heart failure. For example, Jin et al. reported that hypermethylation *ACTB* (β-actin) gene in peripheral blood was associated with increased risk of CAD in male heart failure patients [57]. The study showed that cumulative methylation levels of CpG sites such as *ACTB*_CpG_2.3, ACTB_CpG_7.8, and ACTB_CpG_9.10 were significantly different between patients with CAD, male patients with CAD, and patients with New York Heart Association classes I & II heart failure and CAD, compared to normal controls [57]. Estimating hypermethylation levels of epigenetic markers from peripheral blood samples could be a useful non-invasive method for identifying patients with CAD and heart failure.

Therapeutic agents that alter the expression and function of key genes involved in CAD and heart failure could potentially improve clinical outcomes. For example, Nicholls et al. in a double blinded randomized controlled trial showed that apabetalone decreased hospitalization rates in heart failure patients with type 2 diabetes mellitus and recent onset acute coronary syndrome (ACS) [58]. Apabetalone is a selective inhibitor of bromodomain and extra-terminal (BET) proteins, which are epigenetic regulators of gene expression. However, the potential benefits of apabetalone for treating heart failure in type 2 diabetics with ACS is still unclear. Similarly, a Chinese medicine, Fuzheng Yangxin Recipe (FZTX), was shown to be beneficial in heart failure secondary to ischemic heart disease (IHD). For example, Wang et al. reported that FZTX administered in vivo inhibited apoptosis, enhanced the expression and phosphorylation of signal transducer and activator of transcription 3 (STAT3) gene, leading to improved cardiac functions [59]. These findings show that novel pharmacological agents that target genetic markers could be useful for CAD associated heart failure.

### Hypercholesteremia and lipoprotein abnormalities

Hypercholesteremia is a known risk factor for CAD, which further aggravates the risk for heart failure. A study by Aboumsallem et al. showed that feeding high-sucrose/high-fat diet to C57BL/6J low-density lipoprotein receptor (LDLr)^−/−^ mice resulted in combined hypercholesteremia and type 2 diabetes mellitus, as well as pathological cardiac remodeling [60]. These effects were alleviated following transduction of low-density lipoprotein receptor (LDLr) via adeno-associated virus-8 (AAV-8) in HFpEF model LDLr^−/−^ mice. This led to reduction in plasma cholesterol levels, reversal of heart failure induced pathological cardiac remodeling, and improved treadmill exercise capacity [60]. Similarly, Muthuramu et al. showed that LDLr gene transfer via AAV-8 alleviated pressure overload induced heart failure in LDLr^−/−^ hypercholesteremia model female C57BL/6 mice, following transverse aortic constriction [61]. AAV-8 mediated LDLr transfer led to decreased cardiac remodeling changes such as reduction in left ventricular mass, post-hypertrophic myocardial protein, interstitial fibrosis, and perivascular fibrosis. LDLr gene transfer also significantly decreased plasma cholesterol levels in mice model. These findings show that upregulation of LDL receptors in hypercholesteremia could decrease cardiac remodeling processes, in addition to reducing circulatory cholesterol levels.

Currently, there are several lipid lowering agents, and the most commonly used are statins. Statins are well known for their effects in reducing LDL cholesterol; however, they do not have any effects on lipoprotein a [Lp(a)]. Lp(a), which is encoded by the *LPA* gene is a pro-atherogenic factors that independently increases the risk for cardiovascular events including heart failure [62]. Another drug, alirocumab, which is a proprotein convertase subtilisin/kexin type 9 (PCSK9) inhibitor, decreases both LDL cholesterol and Lp(a) in ACS patients with or without heart failure [63]. This drug significantly reduces major adverse cardiovascular events (MACE); however, it does not have any effect on decreasing hospitalization rates. PCSK9 is a protein secreted by the liver that interacts with LDLr and CD36, promoting their internalization and degradation [64]. Studies have shown that PCSK9 is strongly associated with heart failure pathophysiology. For example, Bouwens et al. showed that temporal increase in PCSK9 levels and decreased LDLr levels were associated with adverse cardiovascular events such as heart failure hospitalizations, left ventricular assist device implantations, heart transplantations, and mortality [65]. Similarly, Bayes-Genis et al. showed that PCSK9 levels had a linear relationship with the risk of mortality among heart failure patients [66]. Laudette et al. reported that cardiomyocyte specific PCSK9 deficient mice prematurely died due to developmental defects that affected cardiac structure and function [67]. This study showed that PCSK9 deficiency resulted in defective cardiomyocyte energy metabolism, mitochondrial metabolism, and electron transport chain complexes [67]. Similarly, Da Dalt et al. showed that PCSK9 deficient mice model had echocardiographic features of HFpEF with defects in cardiac lipid metabolism and mitochondrial functions that were independent of LDLr levels [68]. However, contrary to these findings, Trudsø et al. showed that there were no associations between PCSK9 genetic variants and heart failure that could be implicated to structural and functional cardiac remodeling [69]. This study showed no significant associations between PCSK9 carriers such as loss-of-function variant and R46L missense variant, and left ventricular mass or left ventricular ejection fraction [69].

Mendelian randomization studies conducted using data from the genome wide association studies (GWAS) from the UK Biobank and Heart Failure Molecular Epidemiology for Therapeutic Targets (HERMES) consortium have identified key genetic determinants of lipid marker traits and hypolipidemic drug targets that are associated with heart failure [70]. Genetically derived lipid traits included LDL-cholesterol, triglycerides (TG), HDL-cholesterol and apoprotein B (ApoB), while potential hypolipidemic drug targets included *PCSK9*, *CETP* and *LPL* genes. This study showed that there were substantial genetic associations between these markers and heart failure which were largely mediated by CAD. These findings suggest that PCSK9, which has recently been therapeutically targeted for hyperlipidemia, is an important target for further evaluation in heart failure as well.

### Chronic kidney disease

Patients with chronic kidney disease (CKD) are at a higher risk of developing heart failure, and heart failure could affect renal perfusion and function, thereby increasing the risk for kidney disease. CKD is often associated with cardiac manifestations such as left ventricular diastolic dysfunction (LVDD). However, the mechanisms responsible for cardiac dysfunction in renal impairment are unclear. One possible mechanism could be the epigenetic modifications in the form of enhanced methylation in the vascular endothelial growth factor (VEGF) signaling genes leading to impaired angiogenesis and decreased cardiac microvascular composition [71]. In CKD-LVDD model pigs compared to controls, the expression of the cardiac VEGF signaling gene and VEGF protein was suppressed in association with enhanced methylation, which was linked to a reduction in the subendocardial microvascular density. The cardiorenal syndrome in which both the heart and the kidney affect one another could be due to certain differentially expressed miRNA and genes. Chade et al. showed that alteration in cardiac miRNA and transcriptomic milieu were associated with cardiac remodeling changes such as abnormal diastolic relaxation, mitochondrial injury, moderate LV fibrosis, and myocardial lipid accumulation among swine model of CKD-LVDD [72]. Differential gene expression analysis showed that CKD-LVDD swine models had upregulation of 9 miRNAs and 125 mRNAs and downregulation of 17 miRNAs and 172 mRNAs, compared to controls. This study, using integrated miRNA-/mRNA-seq analysis, found 71 overlapping downregulated mRNA targets of upregulated miRNAs, and 39 overlapping upregulated mRNA targets of downregulated miRNAs in CKD-LVDD swine models, compared to controls. These genes were predominantly involved in cardiac remodeling processes such as ATP and fatty acid production, ubiquitination, and remodeling of the extracellular matrix [72]. Ahmed et al. using two microarray datasets following R/GEO2R analysis found that there were 5 miRNAs (hsa-mir-122-5p, hsa-mir-222-3p, hsa-mir-21-5p, hsa-mir-146a-5p, and hsa-mir-29b-3p) that were involved in cardiorenal syndrome [73]. This study also showed that hsa-miR-21-5p was common in 8 out of the top 10 pathways identified by the enrichment analysis. These findings highlight the importance of molecular changes occurring during the progression of renal disease, which also affects the cardiac tissue. These molecular mechanisms could be possibly targeted for therapeutic interventions in CKD associated heart failure.

Sacubitril and valsartan combination belongs to a new pharmacological class of drugs known as angiotensin receptor neprilysin inhibitor (ARNI), which is approved by FDA for the treatment of heart failure with reduced ejection fraction [74]. Neprilysin inhibition is beneficial in improving cardiac outcomes in patients with chronic renal disease [75]. The United Kingdom Heart and Renal Protection-III trial showed that administration of sacubitril/valsartan did not show any significant improvement in renal outcomes, compared to irbesartan [76]. However, sacubitril/valsartan substantially decreased both systolic and diastolic blood pressures as well as NTproBNP and Troponin I. Another drug, finerenone, which is a third-generation selective mineralocorticoid receptor antagonist has shown protective role in managing HFrEF in patients with concomitant diabetes mellitus and chronic kidney disease [77]. Similarly, Filippatos et al. in a randomized control trial showed that finerenone was effective in reducing primary and recurrent hospitalizations as well as cardiovascular deaths in heart failure patients with CKD and type 2 diabetes mellitus [78]. These findings show that finerenone decreased new-onset heart failure and improved outcomes among existing heart failure patients with CKD and type 2 diabetes. In another study by Filippatos et al. finerenone decreased NT-proBNP levels by 30% or more among patients with worsening HFrEF and CKD and diabetes mellitus [79]. This suggests that specific targeted approaches could improve the heart failure outcome in CKD and other associated co-morbidities.

### Atrial fibrillation

Atrial fibrillation can either be a cause or a result of heart failure and can be managed by both pharmacological and non-pharmacological interventions. Non-pharmacological interventions like catheter ablation have led to decreased mortality, fewer hospitalizations, and lower cardiovascular endpoint events, compared to patients receiving medical treatments [80]. However, even after ablation treatment the success rates are not greater than 50–60% among patients with structural heart disease [81]. Even with extensive linear ablation which could result in decreased atrial contractility and complications, success rates are limited. Considerable research has been dedicated towards understanding the mechanism of atrial fibrillation as the available treatment options are not completely effective. Therefore, understanding the molecular changes in atrial fibrillation is important to decrease the need for cardiac interventions as well as formulating therapeutic strategies.

Our current understanding of the genome wide changes occurring in atrial fibrillation associated heart failure is limited, and further exploration could help in discovering newer pathways and processes for therapeutic interventions. In a study that investigated the gene expression changes from left and right atrial appendages among patients with atrial fibrillation and heart failure, Zeemering et al. identified that 35 genes were significantly altered [82]. This study using gene set enrichment analysis also showed that patients with concomitant atrial fibrillation and heart failure had significantly enriched gene sets for pathways such as cellular respiration, while patients with atrial fibrillation and without heart failure showed enriched gene sets for inflammatory processes. Zhuang et al. using bioinformatic methods found that *FRZB* and *SFRP4* genes were co-upregulated, while *ENTPPL*, *AQP4*, and *C1orf105* genes were co-downregulated among patients with atrial fibrillation and heart failure [83]. These differentially expressed genes were associated with the Wnt signaling pathway, which is related to cardiac fibrosis.

Earlier studies have reported some important genetic associations between atrial fibrillation and heart failure. For example, Amir et al. observed that polymorphism in aldosterone synthase (*CYP11B2*) T-344 C gene was associated with enhanced aldosterone activity, which increased the risk for developing atrial fibrillation among heart failure patients [84]. This study also showed that *CYP11B2* CC genotype was an independent risk factor for atrial fibrillation. In another study among rabbit model, the omega-3 fatty acid, eicosapentaenoic acid, decreased structural remodeling changes associated with atrial fibrillation and heart failure [85]. This study also showed that eicosapentaenoic acid treatment was associated with decreased levels of collagen I and III mRNA and TGF-β1 expression, which were elevated after ventricular tachypacing, which induces heart failure. TGF-β1 is a key molecule responsible for electrical and structural remodeling changes associated with interstitial fibrosis and decreased TGF-β1 levels could be beneficial in preventing these changes. However, Behnes et al. found that TGF-β1 levels could be lower among patients with atrial fibrillation and heart failure and TGF-β1 was inversely correlated with left atrial diameter and NT-proBNP [86]. This could be due to the higher rate of consumption of TGF-β1 within the impaired myocardium or due to autocrine and paracrine antifibrotic effects of natriuretic peptides. In a recent study, Ma et al. reported that intermedin 1–53 (IMD1-53) decreased the occurrence of atrial fibrillation among myocardial infarction induced heart failure rat models by suppressing TGF-β1/Smad3 and TGF-β1/Nox4 related fibrosis [87]. In the Smad-dependent pathway, TGF-β binds to ALK5 and type II TGF-β receptor (Tβ RII), thus creating the Smad 2/3/4 complex, leading to Smad protein-mediated signal transduction and associated fibrosis. TGF- β 1 and Nox4 play a crucial role in regulating the fibrogenesis, which may result in the buildup of collagen, excessive atrial fibroblast proliferation, and aggravated atrial structural remodeling, all of which can finally result in atrial fibrillation. Another mechanism that is associated with arrythmia and heart failure includes inherited mutations in *RYR2* gene and destabilization of its protein product which leads to pathological diastolic Ca^2+^ leak from the sarcoplasmic reticulum [88]. A new class of drugs called rycals that prevent Ca^2+^ leaks from the sarcoplasmic reticulum by interacting and stabilizing the RYR2 channels could be potentially beneficial in managing atrial fibrillation and heart failure [88]. Nofi et al. showed that long-term dantrolene administration to myocardial infarction induced heart failure rat models decreased the thresholds for atrial fibrillation inducibility and alleviated LV dysfunctions [89]. This was associated with lower RYR2 phosphorylation and favorable reprogramming of many altered gene expressions related to sympathetic signaling, ion channels, inflammatory markers, and oxidative stress.

Increased activity of Ca^2+^/calmodulin-dependent protein kinase II (CaMKII) is observed in persistent atrial fibrillation and potentially induces electrophysiological changes in the atrial myocardium [90]. Gene transfer of CaMKII inhibitory peptide (CaMKIIn) into the atrial myocardium of porcine atrial fibrillation-heart failure models inhibited the activity of CaMKII induced electrophysiological and structural changes [90]. CaMKII inhibition conserved atrial contractility and decreased atrial hypertrophy, fibrosis, and apoptosis, but had no effect on inflammation or myolysis. Thus, gene therapy based CaMKII inhibition could be a novel therapeutic approach for decreasing the electrophysiological and structural remodeling associated with atrial fibrillation and heart failure.

### Association between heart failure and neurological diseases

Longer life expectancies have increased the risk for neurodegenerative disorders such as Parkinson’s disease and Alzheimer’s disease. However, the association between heart failure and neurological diseases is not completely understood. Difficulties in pharmacologically treating these disorders have necessitated exploration of the underlying molecular and signaling pathway to device targeted therapy to improve outcomes [91]. For example, in Parkinson’s disease the possible cause for development of cardiovascular morbidities are cardiac dysautonomia, atherosclerosis, insulin resistance, metabolic syndrome, dyslipidemia, and oxidative stress [92]. In addition, certain therapeutic agents used in the management of neurological diseases such as Parkinson’s disease have shown to increase the risk for developing heart failure, especially among patients receiving dopamine agonist such as pramipexole and cabergoline [93]. Cabergoline increased the risk of developing cardiac valve fibrosis thereby aggravating heart failure while the etiology underlying pramipexole is unclear. In a systematic review, Tran et al. reported that dopaminergic agonist pramipexole and cabergoline showed a significantly higher risk for developing heart failure [94]. On the contrary, other medications such as bromocriptine which serves as a partial agonist of D1 dopaminergic receptor and specific agonist on D2 receptor have shown anti-hypertensive and anti-inflammatory effect in patients with Parkinson’s disease, which could have potential role in managing cardiovascular morbidities associated with treatment in Parkinson’s disease [95]. Bromocriptine therapy inhibits sympathetic functions and Na/K ATPase activity, resulting in lower blood pressure levels and decrease circulating levels of pro-inflammatory cytokines such as interleukin (IL)-1B, IL-18, chemokine CCL2, MCP-1, and pro-inflammatory hormone prolactin, thus reducing inflammation. Comorbidities associated with heart failure such as atrial fibrillation could be complicated by Parkinson’s disease. Kincl et al. reported higher prevalence of atrial fibrillation in Parkinson’s disease which was associated with lower blood pressure, lower heart rate, increased cardiac mass as well as right and left ventricular end-systolic and end-diastolic volumes [96]. However, a study using National Inpatient Sample (NIS) database showed that coexisting Parkinson’s disease and atrial fibrillation does not however increase in-hospital mortality and had lower risk of acute heart failure and ventricular tachycardia [97]. In spite of these associations, the molecular mechanisms driving these relationships need to be explored.

Alzheimer’s disease is another debilitating chronic neurodegenerative disorder which is associated with impaired cardiac diastolic function [98]. Possible pathological cues associated with cardiac degeneration in Alzheimer’s disease could be the accumulation of amyloid-β proteins and presenilin (*PSEN*) mutations which are also responsible for reduced cerebral perfusion, resulting in dementia and neurological dysfunction. In the cerebral tissue, aggregation of amyloid-β40 (Aβ40) and amyloid-β42 (Aβ42) plausibly induces inflammatory processes, resulting in cognitive dysfunction. Aβ42 amyloid has particularly shown association with Alzheimer’s disease, while Aβ40 is primarily related to cerebrovascular disease [99]. Intravascularly flowing Aβ amyloid have shown depositions in the vascular and myocardial tissues. Histologically confirmed Aβ amyloids (Aβ40 and Aβ42) related to Alzheimer’s disease were observed in the cardiomyocytes and the cardiac interstitium and were associated with varying levels of myocardial dysfunction. Circulatory levels of Aβ40 shows a positive association with N-terminal pro–B-type natriuretic peptide and high-sensitivity cardiac troponin T in patients not having cognitive impairment and coronary artery disease, indicating possible connection between Aβ40 and myocardial stress [99]. In a meta-analysis, Li et al. reported a positive association between heart failure and risk of all-cause dementia but no significant link between heart failure and Alzheimer’s disease [100]. This necessitated the need for further large-scale studies to identify the potential link between heart failure and Alzheimer’s disease.

### Precision medicine

**Fig. 1:**
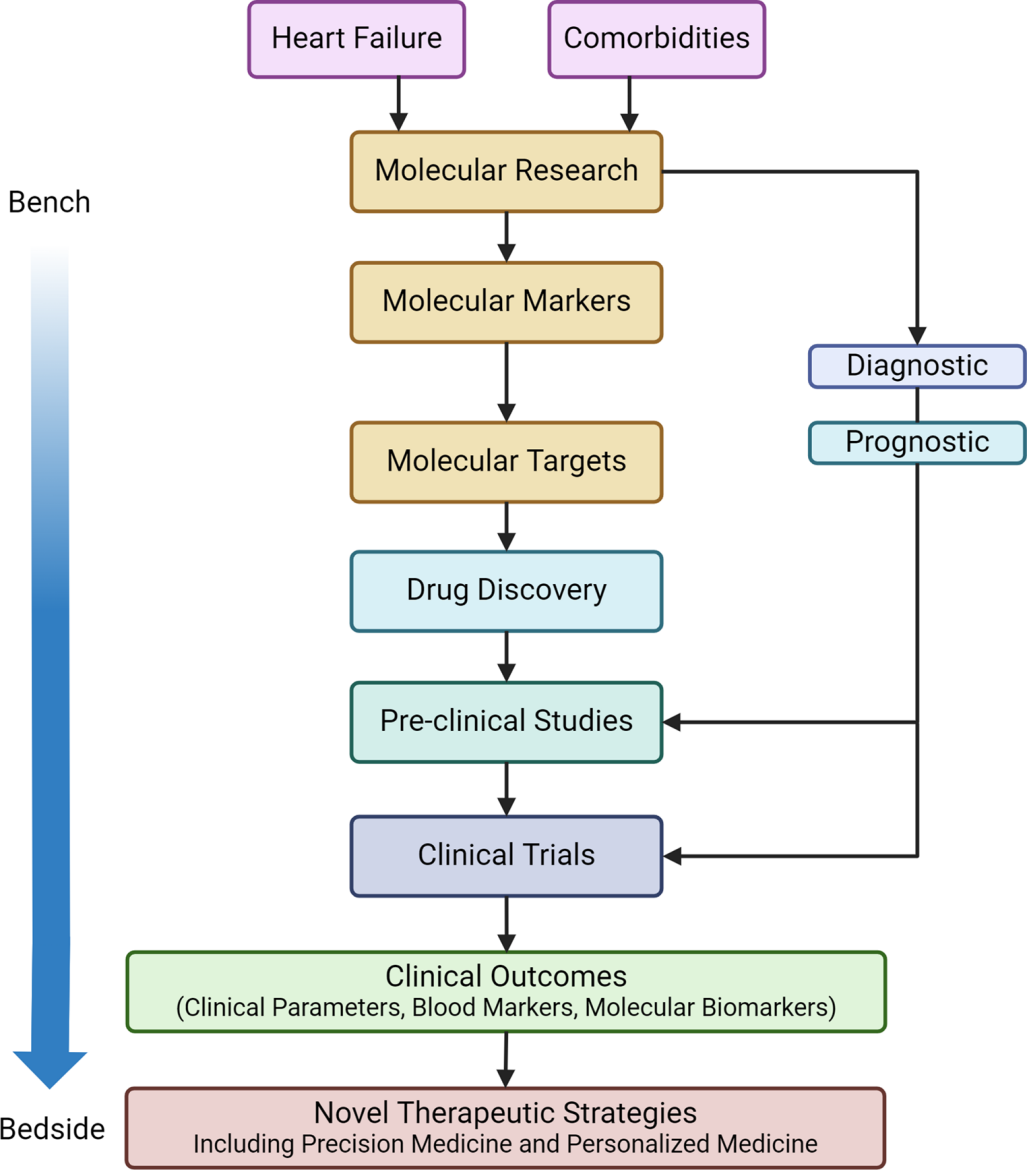
Identifying potential targets for therapeutic approaches that may contribute to bench to bedside approach for clinical management

Heart failure is often associated with comorbidities which further complicate the management and prognosis of the condition. A personalized approach will be more beneficial for the diagnosis, treatment, and prevention of heart failure and its associated comorbidities. One such approach is precision medicine, which aims to tailor medical care to each patient’s individual characteristics, such as genetic makeup, lifestyle, and environmental factors. One of the key features of precision medicine is the use of advanced genomic and molecular techniques to identify genetic variants and molecular alterations affecting key pathways that influence disease susceptibility, progression, and response to therapy. Several studies, as discussed above, have identified genetic loci and molecular pathways associated with heart failure and its comorbidities. Table 1 shows the list of key molecular alterations occurring in specific comorbidities in heart failure and their significance. These genetic variants and molecular alterations could have functional implications for the underlying molecular pathways involved in the pathogenesis of heart failure and comorbidities. These are potential targets for therapeutic approaches that may contribute to translation of these discoveries from bench to bedside for clinical management (Fig. 1).

**Table 1:** List of key molecular alterations occurring in specific comorbidities in heart failure and their significance

Author and year	Comorbidity in heart failure	Molecular alteration	Significance/outcome
Hahn et al. 2021 [11]	Obesity	Upregulated genes in HFpEF	Enriched in pathways of mitochondrial adenosine triphosphate synthesis/electron transport
		Downregulated genes in HFpEF	Enriched in pathways related to endoplasmic reticulum stress, autophagy, and angiogenesis
Satoh et al. 2021 [11]	Obese metabolic syndrome HFpEF model rats	↓ sGCβ1	Enhanced exercise induced pulmonary arterial hypertension and ROS production
		↑ miR-193b	Enhanced degradation of NFYA which eventually reduces sGCβ1
Mishra et al. 2021	Obesity	↓ cyclic GMP sensitive phosphodiesterase 9	Decreased fat deposition and improved left ventricular function associated with PPARα upregulation
Schiattarella et al. 2021 [12]	Obesity	↑ Xbp1s	Suppress myocardial lipid accumulation in HFpEF by modulating unfolded protein response and increasing degradation of FoxO1
Almengló et al. 2022 [14]	Obesity	↑ neutrophilic CD11b	Adipose tissue derived secretome increased neutrophilic infiltration via CD11b while SGLT2 inhibitor dapagliflozin mitigated it
Wang et al. 2023 [13]	High fructose diet in vivo/obesity model	↑ MCP-1	Increased expression of pro-fibrotic genes and inflammatory mediators associated with cardiac macrophage infiltration
Han et al. 2022 [23]	Diabetic cardiomyopathy model	↑ PI3K/AKT/mTOR/HIF-1α ↑ NOX2 and NOX4 ↓ miR-372-3p (after Knock down)	Enhanced angiogenesis in vivo and reduces risk for diabetic cardiomyopathy
Wu et al. 2022 [24]	Diabetes in HFpEF model	↑ SIRT6	Repressed endothelial PPARγ expression which mitigated endothelial fatty acid uptake
Li et al. 2019 [26]	Diabetes mellitus	↑ miR-320	Enhance the expression of fatty acid translocase resulting in augmented cardiac lipid uptake and cardiomyocyte lipotoxicity
Veitch et al. 2022 [27]	Diabetes mellitus	↓ miR-30 microRNA members	Alleviates oxidative stress and endothelial cell senescence
Mu et al. 2020 [28]	Diabetes mellitus	↑ BRD4	Reduces PINK1/Parkin-mediated mitophagy leading to accumulation of damaged mitochondria and induces cardiac dysfunction
Xu et al. 2022 [31]	Diabetes mellitus	MAPK1, ARRB1 and ABL1	Potential targets of calycosin in HF with diabetes mellitus
Pattathu et al. 2016 [46]	Idiopathic PAH	*BMPR2* mutation	Commonly associated with PAH
Du et al. 2022 [46]	CHD-PH	*BMPR2* mutation	Cardiac dysfunction and poor calcium transients
Houssaini et al. 2013 [49]	PH	↑ mTORC1 ↑ mTORC2	Induced remodelling in pulmonary vasculature which was reversed by rapamycin
McNair et al. 2021 [50]	HH induced PH model	Rapamycin mediated mTOR ↓	Unable to reduce PH and RVF in older mice due excessive loss of body weight
Legchenko et al. 2018 [52]	PH with RVF model	↑ pre-miR-197 ↑ pre-miR-146b	Under expressed genes such as *Cpt1b* and *Fabp4* leading to impaired fatty acid oxidation
Frump et al. 2021 [53]	PAH	↑ Apelin	Stimulated by 17β-estradiol and alleviates PAH induced RV injury through BMPR2 mediated signalling
Shou et al. 2022 [35]	Hypertensive model	↓ PINK1	Suppressed Drp1^S616^ phosphorylation resulting in impaired mitochondrial fission
Bednarski et al. 2022 [36]	Spontaneous hypertensive model	↓ AMPK ↓ PPAR-α	Impaired cardiac lipid metabolism characterized by triacylglycerol accumulation and reduced β-oxidation
de Couto et al. 2022 [38]	HFpEF model hypertensive rats	↑ eNOS ↓ VCAM-1	CDC mediated vasodilation and reduced monocyte function in vitro
Zhang et al. 2022 [39]	Hypertensive cardiac hypertrophy model	↑ *Eln* ↑ *Tgfb3*	Positively correlated with the disease severity of experimentally induced cardiac hypertrophy in mouse model
Ren et al. 2021 [41]	Essential hypertension	*WNT3A* rs752107(C > T) polymorphism	*WNT3A* rs752107 TT genotype has higher risk for essential hypertension as compared to CC variant
Kravchun et al. 2020 [55]	CAD and obesity	*IL6* gene polymorphism	-174G allele and GG genotype have higher risk of developing HF
Jin et al. 2022 [57]	CHD	*ACTB* (β-actin) hypermethylation	Linked with HF and male subjects in CHD
White et al. 2022 [63]	ACS	PCSK9 inhibition	PCSK9 inhibitor alirocumab reduced LDL cholesterol and Lp(a) as well as MACE but did not reduce HF hospitalization
Aboumsallem et al. 2019 [60]	T2DM and Hypercholesteremia	↑ LDLr via AAV-8 mediated gene transfer	Reduced plasma cholesterol, reversed HF induced cardiac remodeling and improved exercise performance in LDLr^−/−^ mice
Muthuramu et al. 2019 [61]	Hypercholesteremia	↑ LDLr via AAV-8 mediated gene transfer	Reduced cardiac remodelling in LDLr^−/−^ mice
Da Dalt et al. 2021 [68]	Cardiomyocyte lipid metabolism in vivo	PCSK9 inhibition in vivo	Altered cardiac lipid metabolism and defective mitochondrial function associated with progression to HFpEF
Eirin et al. 2022 [72]	CKD-LVDD model	↓ VEGF signalling genes	Reduced VEGF expression and subendocardial microvascular density
Chade et al. 2022 [72]	CKD-LVDD model	↑ Cardiac remodelling genes	Genes involved in ubiquitination, ATP and fatty acid production, and extracellular matrix remodelling
Zhuang et al. 2022 [83]	Atrial fibrillation	↑ *FRZB*, *SFRP4* ↓ *ENTPPL*, *AQP4*, *C1orf105*	Differentially expressed genes related to the wnt signalling pathway
Ma et al. 2023 [87]	Atrial fibrillation in MI induced HF model	↓ TGF-β1/Smad3-related fibrosis ↓ TGF-β1/Nox4 activity	Mechanism for intermedin 1–53 mediated in vivo suppression of atrial fibrillation
Nofi et al. 2020 [89]	Atrial fibrillation	↓ RYR2 phosphorylation	Reduced AF inducibility in HF and reduced left ventricular dysfunction
Liu et al. 2019 [26]	Porcine AF-HF model	CaMKIIn gene transfer	Gene therapy mediated CaMKII inhibition mitigated cardiac remodelling changes

Abbreviations: AAV-8, adeno-associated virus-8; ABL1, Abelson murine leukemia viral oncogene homolog 1; ACS, acute coronary syndrome; AF, atrial fibrillation; ARRB1, arrestin beta 1; ATP, adenosine triphosphate; *BMPR2*, bone morphogenetic protein receptor type II; CaMKIIn, Ca^2+^/calmodulin-dependent protein kinase II inhibitory peptide; CAD, coronary artery disease; *Cpt1b*, carnitine palmitoyltransferase 1B; CHD, coronary heart disease; CHD-PH, congenital heart disease associated PH; CKD, chronic kidney disease; *Eln*: tropoelastin; eNOS, endothelial nitric oxide synthase; *Fabp4*, fatty acid binding protein 4; HF, Heart failure; HFpEF, Heart failure with preserved ejection fraction; HH, hypobaric hypoxia; *IL6*, Interleukin-6; LDL, low-density lipoprotein; LDLr, low-density lipoprotein receptor; LVDD, left ventricular diastolic dysfunction; MACE, major adverse cardiovascular events; MAPK1, mitogen-activated protein kinase-1; MCP-1, monocyte chemoattractant protein-1; mTORC1, mammalian target of rapamycin complex 1; mTORC2, mammalian target of rapamycin complex 2; NFYA, nuclear factor Y α subunit; PCSK9, proprotein convertase subtilisin/kexin type 9; PI3K, phosphoinositide 3-kinase; PINK1, PTEN-induced kinase 1; PPARα, peroxisome proliferator-activated receptor alpha; ROS, reactive oxygen species; RV, right ventricle; RVF, right ventricular failure; RYR2, Ryanodine Receptor 2; sGCβ1, soluble guanyl cyclase beta 1; *Tgfb3*, transforming growth factor beta 3; VCAM1, vascular cell adhesion molecule 1

Symbols: ↑-upregulated/overexpressed/elevated; ↓-downregulated/under-expressed/suppressed

## Conclusion

Heart failure and associated comorbidities present a significant clinical challenge, as they are multifactorial and often complicate one another. Our review provides an overview on the current research in molecular mechanisms associated with heart failure and comorbidities and provides critical insights about the underlying pathophysiology of these conditions. However, some of these findings are observed in animal studies and need to be confirmed in humans. Nonetheless, this knowledge could lead to the identification of novel targets for drug development and more effective and safer therapies. This knowledge is also critical for developing precision medicine strategies to improve the outcomes for patients living with these conditions.

## Data Availability

No datasets were generated or analysed during the current study.
